# Principles of Organic and Physiological Chemistry

**Published:** 1854-01

**Authors:** 


### REVIEW DEPARTMENT.

### ARTICLE X.

*Principles of Organic and Physiological Chemistry.* By CARL LÖWIG, Doctor of Medicine and Philosophy, ordinary Professor of Chemistry in the University of Zurich, &c. Translated by DANIEL BREED, M. D., of the U. S. Patent Office, &c. Philadelphia, A. Hart, late Carey & Hart, 1853.

THE work before us is a laudable, laborious and learned attempt to introduce something like order and system into that great chaos of facts which we call organic chemistry. If there be any domain of science from which the student shrinks appalled, at the magnitude of the field and the multitude of the facts which throng it, it is this. A thousand acute and untiring observers have for these many years been laboring here. Every root, every flower, every leaf in the vegetable kingdom, every secretion, every tissue of the animal world has been scrutinized, peered into by microscopes and polariscopes, tortured with all imaginable acids, alkalies and salts, charged with galvanism, subjected to all temperatures. From these manipulations have arisen a host of new compounds.

Isis refuses to have her veil lifted, Proteus flows away under a thousand shapes. But vain are all his disguises, his pertinacious pursuers have more powerful incantations than the ancient shepherds that caught him on the shore. Every new guise he assumes is carefully copied and elaborately studied.

To all these hosts of new compounds, modern science has given names as repulsive to the novice as Almyzinthra, the Green Dragon, and the other mystic titles of the old quackish fathers of chemistry. Alchemy itself can boast of few more embarrassing and unintelligible words than *mercaptan, benzidam trinitranisol, kapnomur*, or a thousand others which modern chemistry has introduced. To add to the confusion resulting from such barbarous words, they have hitherto been scattered pretty much at random through our chemical works. The feeblest possible tie is sufficient to hold them in juxtaposition; the merest accident has served as a basis of classification.

Dr. Löwig has taken up this unpromising subject. He has attempted to coerce this anarchical mob into regular battalions, and to classify them behind their appropriate banners. Like all first classifiers he has probably laid himself open to the charge of too great artificiality in his arrangement. But surely an artificial classification is better than none at all. It is an aid to the memory. It exhibits to us some points of resemblance among these numerous isolated individuals. It enables them to group themselves so that we can glance along the ranks and rectify defects afterwards. If Linnæus had not made his artificial system, it may be doubted whether we should at this day so greatly reverence Jussieu and Cuvier.

Löwig regards this book as an introduction to his larger work, the Chemistry of Organic Combinations. This will account for the absence of certain facts. Thus, he neither states the name of the discoverer of an organic compound, nor does he give the per centage of its components, but confines himself wholly to the expression of chemical composition by formulæ.

Certain organic bases have always been admitted. Thus, acetic acid $C_4\ H_3\ O_3$ has for some time been written in this manner: $(C_4\ H_3)\ O_3$, $C_4\ H_3$ being taken as the organic basis acetyl. This is no hypothesis but an actual truth susceptible of demonstration, for if chlorine, bromine or any other element be made to decompose acetic acid, we find that each element enters as three atoms which take the place of the three atoms of oxygen, giving as such compounds as $(C_4\ H_3)\ Cl_3$, $(C_4\ H_3)\ Br_3$, &c. This may be regarded as a matter already established.

But this has left the science of chemistry encumbered with a confused rabble of bases without order, proportion or symmetry, a great mob of *proximate elements*, (to use a very common but very questionable phrase.) It, therefore, remained an open question, how the elements in these radicals were themselves combined. It was to this department of inquiry that Löwig directed his attention. He found that a very simple law of increase regulated the formation of these radicals, that groups of them differed from each other, in fact, only by a + or — $C_2$ $H_2$. Thus, if from ethyl, $C_4$ $H_5$, $C_2$ $H_2$ be withdrawn, we have methyl, $C_2$ $H_3$; and, if from acetyl, $C_4$ $H_5$, $C_2$ $H_2$ be subtracted, we get formyl, $C_2$ H. Certain other radicals, as benzid, are found to have the same ascending $C_2$ $H_2$, but with the addition to each member of $C_8$, which is regarded as the nucleus of the compound radical.

These preliminary observations being made, it is easily to understand this system of classification, which possesses the advantages of facility of acquisition, clearness and comprehensiveness, so that the relations of these radicals can be retained without unnecessarily burdening the memory.

The *first* and simplest class of radicals, Löwig calls *carbyls.* They consist each of several atoms of carbon, which form the entire base. Thus oxalic acid, $C_2$ $O_3$, is assumed to be the teroxyd of a radical consisting of two atoms of carbon ($C_2$= oxotyl.)

The *second* division comprises those which contain, in addition to the carbon, certain atoms of hydrogen. Acetyl, $C_4$ $H_3$, is an example.

The *third* class embraces the *azocarbyls*, which consist of nitrogen and carbon, as *paraban*, $N_2$ $C_6$.

The *fourth* class includes the *hydroazocarbyls*, or radicals, consisting of nitrogen, hydrogen and carbon. Uren, N $C_2$ H, is an example.

The ammonias are classified separately as *hydryls.*

These, with their *derived* and *paired* radicals, constitute the great majority of the organic bases with which we have to do. By the doctrine of pairing, he explains very clearly, many of those remarkable instances of isomerism which have given chemists so much trouble.

The best illustration of our author's method of dealing with the abstruse inquiries he has undertaken, is to be found in his analysis of his group of *hydrocarbyls*. We have already shown how from ethyl, by the abstraction of $C_2 H_2$, methyl arises ($C_2 H_3$.) If we further subtract this from methyl we get a single atom of hydrogen. We may therefore write out these radicals thus:

*Methyl*, $= C_2 H_3 = C_2 H_2$, H.

*Ethyl*, $= C_4 H_5 = 2 C_2 H_2$, H.

*Amyl*, $= C_{10} H_{11} = 5 C_2 H_2$, H.

Thus we see that these radicals can be divided into two parts, the $C_2 H_2$ or its multiple and the constant nucleus H.

An examination of formyl, acetyl, &c., will lead to a similar result. We have already shown how formyl is obtained by abstracting $C_2 H_2$ from acetyl, and here the subtraction stops, for we have reached $C_2$ H, which does not contain $C_2 H_2$. If we therefore proceed to construct a series, as before, we get the following:

Formyl, $C_2$ H.
Acetyl, $(C_4 H_3) = (C_2 H_2) + C_2$ H.
Propionyl, $(C_6 H_5) = 2 (C_2 H_2) + C_2$ H.
Butyryl, $(C_8 H_7) = 3 (C_2 H_2) + C_2$ H, &c.

An analysis, therefore, of these two groups, gives us the same $C_2 H_2$, or its multiple, in both, with an additional element, which differs in the two series. The group of radicals possessing a positive character, have H added to the $C_2 H_2$, while those which are negative have $C_2$ H. These, therefore, H and $C_2$ H, constitute the *active element* or *molecule*, and the $C_2 H_2$, multiplied by various numbers, the *ascending passive component*.

There is still another series of radicals belonging to the class of hydrocarbyls, differing from both the former. Thus, if from camphyl $C_{10} H_7$ we substract $2 (C_2 H_2)$ we get terecryl $C_6 H_3$. If to the same we add $13 (C_2 H_2) = C_{26} H_{26}$, we get oleyl, $C_{36} H_{33}$, the radical of oleic acid. By a careful examination of the structure of these compounds, we learn that we have here an ascending series similar to that of the formyl group, with this difference, that an additional $C_2$, which is called a nucleus, is introduced into each member. We get, therefore, the following series:

Terecryl, $C_6\ H_3 = (C_2\ H_2)\ C_2\ (C_2\ H.)$

Camphyl, $C_{10}\ H_7 = 3\ (C_2\ H_2)\ C_2\ (C_2\ H.)$

Moringyl, $C_{30}\ H_{27} = 13\ (C_2\ H_2)\ C_2\ (C_2\ H.)$

Oleyl, $C_{36}\ H_{33} = 16\ (C_2\ H_2)\ C_2\ (C_2\ H,)$ &c.

It will be seen that these two series differ from one another in the proportion which the atoms of carbon sustain to those of hydrogen in these two classes of hydrocarbyls. In the one class, which is made up of the formyl and methyl groups, we find that the atoms of carbon differ from those of hydrogen by only one. In the other, there are several atoms of carbon more than of hydrogen in each member. Hence, a division of the *hydrocarbyls* into *hydrisocarbyls*, or those in which the hydrogen and carbon are combined in nearly equal proportions, and *hydropolycarbyls*, or those in which the atoms of carbon considerably exceed those of hydrogen.

From these are derived several other groups, as the *elayl* and *acetonyl groups*, arising from a pairing of the radicals of the formyl and methyl groups, the *succyl* group, a pairing of the oleyl and formyl groups, &c.

By this classification we can distribute a very large number of radicals under appropriate heads. There still, however, remains a great number of animal and vegetable compounds of higher organization, as well as certain products of decomposition, which are not to be ranged in any of these classes. These are treated of under appropriate heads, corresponding to the old distribution of these same compounds.

Whatever may be the ultimate fate of this system, it cannot be denied that, as it is the first, so it is a very successful attempt to give a clear idea of the relations of these numerous compounds. The chemist will find this book altogether indispensable, and will look forward with anxiety for the appearance of the more extended survey of organic chemistry, which has so masterly an introduction. P.

